# Supplementary material for: Covalent organic framework membranes for efficient separation of monovalent cations
Source: Nat Commun. 2022 Nov 19;13:7123. doi: 10.1038/s41467-022-34849-7 (PMC9675805; doi:10.1038/s41467-022-34849-7)
Supplement: Supplementary file 1 — Supplementary Information [file 41467_2022_34849_MOESM1_ESM.pdf]

Supplementary Information for

Covalent Organic Framework Membranes for Efficient Separation of Monovalent Cations

Hongjian Wang, Yeming Zhai, Yang Li, Yu Cao, Benbing Shi, Runlai Li, Zingting Zhu, Haifei Jiang, Zheyuan Guo, Meidi Wang, Long Chen, Yawei Liu, Kai-Ge Zhou, Fusheng Pan*, and Zhongyi Jiang*

*Correspondence to: zhyjiang@tju.edu.cn and fspan@tju.edu.cn

This document file includes:

Supplementary Fig. 1-22

Supplementary Table 1-6

Supplementary Reference 1-12

1. Design and synthesis of phosphoric acid diamine (Pa-PO_3H_2)

In our study, chemical functionality on COFs was carried out by varying the functional groups (side chains) on diamine linker. The sulfonic acid and carboxylic acid COFs were reported and the corresponding diamines were commercially available. However, the phosphoric acid COF (TpPa-PO_3H_2) and phosphoric acid diamines (Pa-PO_3H_2) have not been reported. Here, to graft -PO_3H_2 group on COFs, we designed phosphoric acid diamines using the recipe listed in Supplementary Fig. 1. Briefly, the synthesis of phosphoric acid diamines could be divided into the following two steps: A mixture of 4.0 mmol of 2-bromobenzene-1,4-diamine, 4.4 mmol of diethyl phosphite ($(EtO)_2P(O)H$), 4.4 mmol of trimethylamine and 0.2 mmol of tetrakis(triphenylphosphine)palladium(0) (tetrakis($(Ph)_3P$)Pd(0)) in 1 mL of anhydrous toluene was added into the reaction tube sealed with a rubber stopper. The reaction tube was treated with three cycles of frozen-degassed operations to maintain N_2 atmosphere and then transferred into oil bath of 90 ^oC for 24 h. After cooling to room temperature, the organic solvent was removed by spin dryer. The resultant product was then purified with preparative thin-layer chromatography (dichloromethane/petroleum ether: 1/1) and dried to give 0.4 g of 2,5-diaminobenzene diethylphosphite as a brown liquid (40%). Afterwards, 0.18 mmol of 2,5-diaminobenzene diethylphosphite was added into 5 mL of anhydrous acetonitrile. The solution was transferred into a glass tube sealed with rubber stopper. Under N_2 atmosphere, 0.88 mmol trimethylsilyl bromide (TMS-Br) was dropped into the glass tube and then the solution was stirred at room temperature for 24 h. The resultant mixture was added with methanol to quench the reaction and then spin-dried to obtain a solid product. The product was then partitioned between 20 mL of diethyl ether and 20 mL of deionized water. The aqueous phase was concentrated and recrystallized in acetone to give a white solid product of hydrated product (2,5-

diaminobenzene diethylphosphite) with a yield of 38%. Structure characterizations of 2,5-diaminobenzene diethylphosphite were carried out using ^{1}H NMR spectroscopy, ^{31}P NMR spectroscopy and ultra-performance liquid chromatography-triple quadrupole tendem mass spectrometry, respectively. ^{1}H NMR spectrum of the product was shown in Supplementary Fig. 2. The product presented a characteristic signal with integral ratio of 1:1:1 at 7.5 p.p.m., 7.2 ppm and 7.0 ppm, which belonged to the three H environments on the benzene ring. At the same time, the characteristic signal with integral ratio of 2:3 could be observed at 3.3 ppm and 1.2 ppm, corresponding to the residual ethyl phosphate H environment after partial hydrolysis in the phosphoric acid group, which indicated that the product was a mixture of fully hydrolyzed/partially hydrolyzed phosphoric acid diamines. The mass spectrometry data of the product further confirmed our conclusion, as shown in Supplementary Fig. 3. The product showed characteristic signals at 187.98 m/Z and 215.02 m/Z, belonging to fully hydrated and partially hydrated phosphoric acid diamine monomers, respectively. In this study, the phosphoric acid COFs nanosheets/powders would be subsequently synthesized under acidic or alkaline conditions, which also provided hydrated conditions for partially hydrolyzed phosphoric acid diamines simultaneously. Therefore, the fully hydrolyzed/partially hydrolyzed phosphoric acid diamine precursors were directly used for the next synthesis process of COF nanosheets or powders. In addition, ^{31}P NMR was used to analyze the monomer product, and it was found that the product exhibited a signal concentrating on 5.06 ppm, verifying the existence of phosphoric acid group on benzene ring (Supplementary Fig. 4).

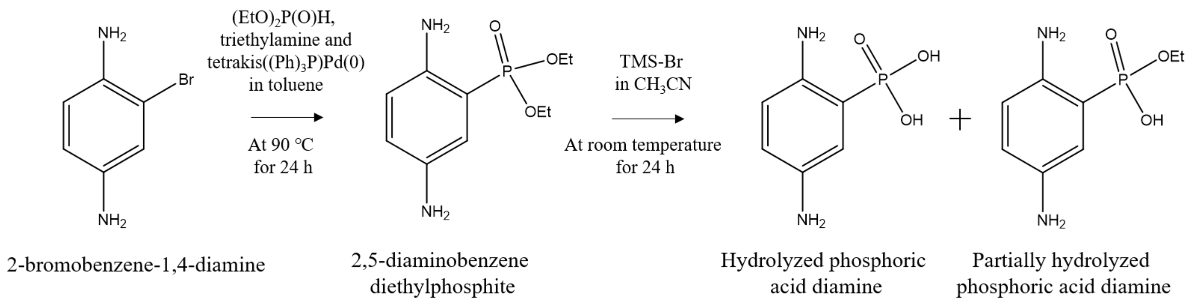

Supplementary Fig. 1 Scheme illustration of synthesis process for phosphoric acid diamines.

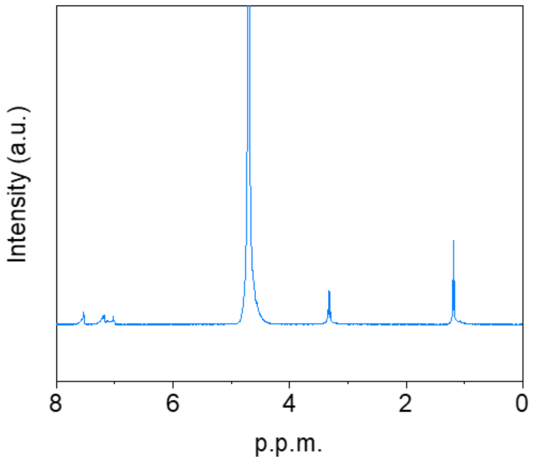

Supplementary Fig. 2 ^{1}H NMR spectra of phosphoric acid diamines.

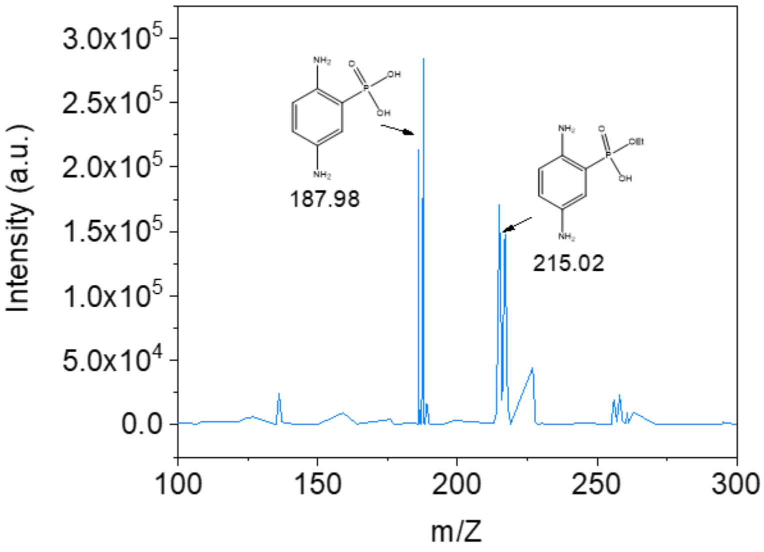

Supplementary Fig. 3 Mass spectra of phosphoric acid diamines.

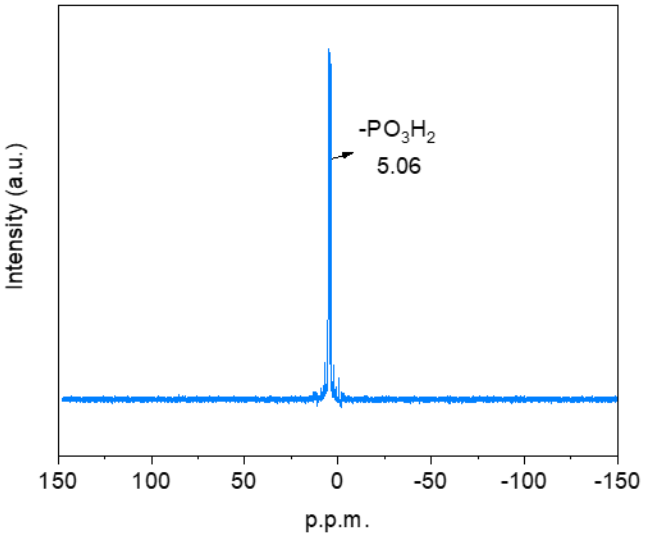

Supplementary Fig. 4 ^{31}P NMR spectrum of phosphoric acid diamines.

2. Characterizations of COF nanosheets

2.1. AFM and TEM

The morphologies of COF nanosheets were analyzed by atomic force microscopy (AFM, Dimension Icon, Bruker, Germany) using 5-Megapixel digital camera with XY scan range of 10 μm × 10 μm, Z range of 10 μm and vertical noise floor < 30 pm RMS. In addition, the morphologies and composition of the COF nanosheets were investigated by high resolution transmission electron microscopy images (HRTEM) using JEOL JEM-F200 microscope with accelerating voltage of 200 KV and STEM resolution ≤ 0.16 nm. Fig 2C-D verified the well-grown lamellar morphologies of TpPa-PO_3H_2 nanosheets. Elemental mapping results further manifested the even distribution of component C, N, O and P on the skeletons of TpPa-PO_3H_2 nanosheets (Supplementary Fig. 5).

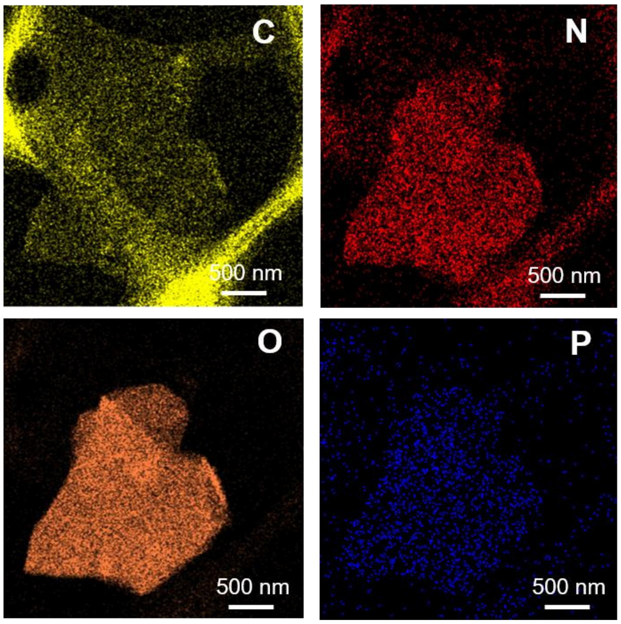

Supplementary Fig. 5 Elemental distribution of C, N, O and P in TpPa-PO_3H_2 nanosheets.

The morphologies of TpPa-SO_3H nanosheets were shown in Supplementary Fig. 6. TpPa-SO_3H nanosheets had a good dispersion in water and exhibited obvious Tyndall effect (Supplementary Fig. 6a). AFM image displayed that the lateral size and thickness of TpPa-SO_3H nanosheets was 5.0-10 μm and 5.0-6.0 nm, respectively (Supplementary Fig. 6b). HRTEM images further confirmed the lamellar structure of TpPa-SO_3H nanosheets, showing a wide banded morphology in Supplementary Fig. 6c. Moreover, the crystalline nature of TpPa-SO_3H nanosheets was investigated by high-resolution TEM images and selected area electron diffraction (SAED) results. It was found regularly arranged lattice fringes (Supplementary Fig. 6d) and arrayed diffraction pots corresponding to the (100) plane vector of 0.35 $Å^{-1}$ (Supplementary Fig. 6e) in the skeletons, manifesting the high crystallinity of TpPa-SO_3H nanosheets. Elemental mapping measurement further indicated that the C, N, O and S elements were evenly distributed within TpPa-SO_3H nanosheets (Supplementary Fig. 6f).

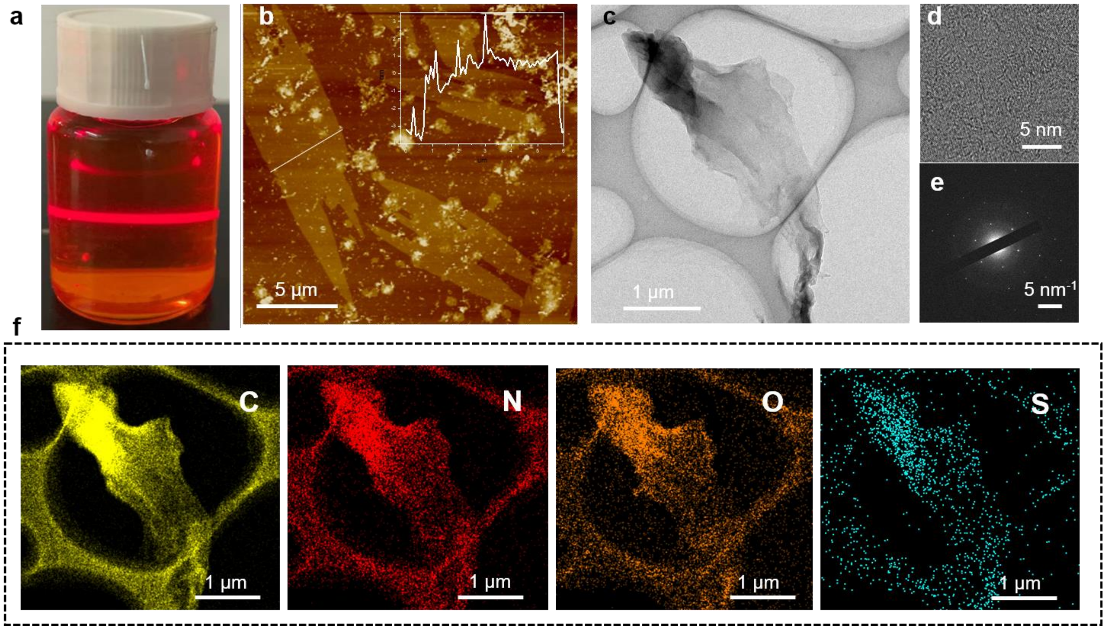

Supplementary Fig. 6 Morphologies of TpPa-SO_3H nanosheets. (a) Nanosheet dispersion of TpPa-SO_3H. **(b)** AFM images, **(c-d)** HRTEM images, **(e)** SAED image and **(f)** elemental distribution images of TpPa-SO_3H nanosheets.

The morphologies of TpPa-CO_2H nanosheets were shown in Supplementary Fig. 7. TpPa-CO_2H nanosheets had a good dispersion in water and exhibited obvious Tyndall effect (Supplementary Fig. 7a). It was observed that the average size of TpPa-CO_2H nanosheets was around 1.5-2.5 μm and the corresponding thickness was ~8.0 nm, as shown in Supplementary Fig. 7b-c. Horizontally aligned lattices were found in TpPa-CO_2H nanosheets (Supplementary Fig. 7d) and uniform diffraction spots corresponding the vectors of 1.9 $Å^{-1}$ for (001) plane were observed in Supplementary Fig. 7e, which indicated the well-defined crystalline structures of TpPa-CO_2H nanosheets. Elemental mapping measurement demonstrated that the C, N, O elements were evenly distributed within TpPa-CO_2H nanosheets (Supplementary Fig. 7f).

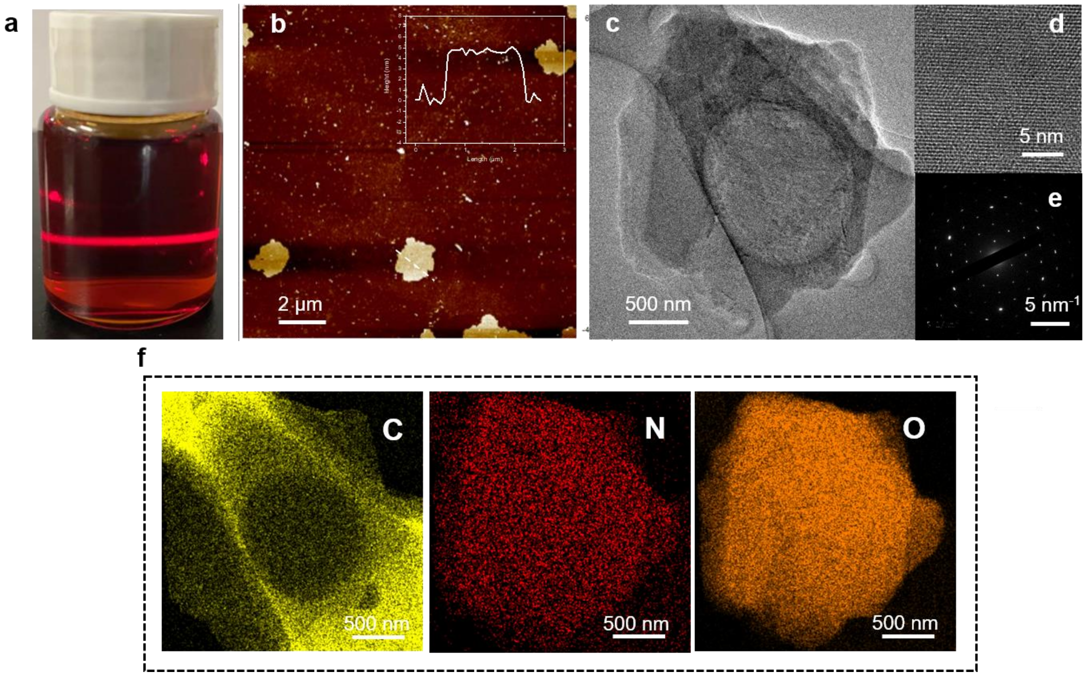

Supplementary Fig. 7 Morphologies of TpPa-CO_2H nanosheets. (a) Nanosheet dispersion of TpPa-CO_2H. **(b)** AFM images, **(c-d)** HRTEM images, **(e)** SAED image and **(f)** elemental distribution images of TpPa-CO_2H nanosheets.

2.2. Formation mechanism of COF nanosheets

Fabricating COFs into nanosheets with high aspect ratio is a persistent hot-topic for constructing continuous COF membranes with tunable structures. To engineer COF nanosheets, the key issue is to accelerate in-plane growth (or heteroepitaxial growth) of COF domains and simultaneously prevent COF domains from aggregation-precipitation. Our previous work has demonstrated manipulation of COF domain-solvent interactions is an alternative to fabricate COF nanosheets[1]. Intensifying COF-solvent interaction to exceed the interaction energy between COF-COF domains would lead to the improved in-plane growth of COF layers and contribute to high quality COF nanosheets. In this study, we manipulated the electrostatic interaction in aqueous phase during tri-phase interfacial polymerization. Taking TpPa-PO_3H_2 as the example, we controlled water phase additives in the buffer layer and modulated the degree of dissociation of phosphoric acid groups in COF side chains, in order to probe the nanosheet forming process. We used 3 M acetic acid, deionized water and 0.5 M sodium bicarbonate aqueous solution as the buffer layer respectively to proceed the interfacial polymerization toward TpPa-PO_3H_2 nanosheets. It could be seen that a large amount of dark-red precipitates appeared in the organic phase when using 3 M acetic acid and deionized water as the aqueous layer. The resultant COFs presented as cluster of aggregates (Supplementary Fig. 8a-b). In contrast, no precipitates could be found and the aqueous phase exhibited obvious Tyndall effect when using 0.5 M sodium bicarbonate aqueous solution as the buffer layer. The resultant COFs showed lamellar morphologies with high aspect ratio (Supplementary Fig. 8c).

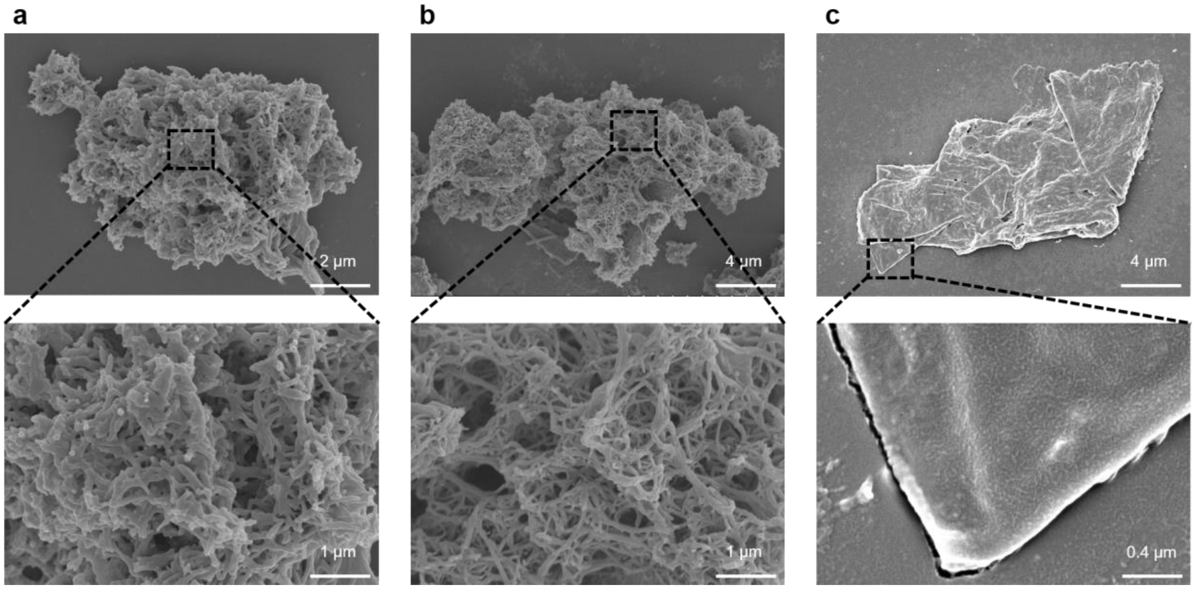

Supplementary Fig. 8 SEM images of TpPa-PO_3H_2 obtained from tri-phase interfacial polymerization using different aqueous buffer. (a) 3 M acetic acid, **(b)** deionized water and **(c)** 0.5 M sodium bicarbonate.

To gain insight into the formation mechanism, we used DFT to optimize the structure of COF layers and calculate the interaction energy. To assess the exchange-correlation energy, generalized gradient approximation with Perdew–Burke–Ernzerhof functional (GGA-PBE) was used. Based on ultrasoft plane-wave, the energy cutoff applied in this work was 500 eV. The energy and force convergence threshold were set to 1.0×10^{-5} eV and 0.01 eV Å^{-1}, respectively. The binding energy (E_b, eV) of COF_{layer1}-COF_{layer2} was calculated using Supplementary equation 1 and listed in Supplementary Table 1.

$$E_b = E_{total} - (E_{COF_{layer1}} + E_{COF_{layer2}}) \quad (1)$$

Where E_{total} (eV) is the total energy of interacted two COF layers, $E_{COF_{layer1}}$ and $E_{COF_{layer2}}$ (eV) correspond to the energy of individual COF layer. As shown in Supplementary Table 1, the lamellar interaction energies under the three conditions are 0.339 eV, 0.911 eV and 1.351 eV

respectively, indicating that the electrostatic interaction between lamellar increased with the dissociation of side chain group being intensified. This trend further revealed the nanosheet formation mechanism: When using 3M acetic acid and deionized water as buffer phase, the phosphoric acid group had a lower degree of dissociation. This was not beneficial to the in-plane growth of the COF nanosheets, thus resulting in the formation of precipitates. In contrast, the introduction of a weak base (e.g., sodium bicarbonate) aqueous solution as buffer aqueous phase could drive the dissociation of phosphoric acid groups in COF units and fortify the interaction between the COF units, thus promoting the two-dimensional growth of the lamellar products and obtaining high quality nanosheets.

Supplementary Table 1. Binding energy of COF-COF interlayers

**COF-COF interlayers**	**Binding energy (eV)**
**TpPa-PO_3H_2–TpPa-PO_3H_2**	0.339
**TpPa-PO_3H_2–TpPa-PO_3H^-**	0.911
**TpPa-PO_3H^-–TpPa-PO_3H^-**	1.351

3. Characterizations of COF membranes

3.1. SEM

SEM characterizations were performed using Apreo S LoVac equipped with Schott airfield firing electron gun. As shown in Supplementary Fig. 9, the well-dispersed COF nanosheets in aqueous solutions enabled the formation of stable and uniform membrane structures. TpPa-SO_3H and TpPa-CO_2H membranes exhibited dark-red and brown morphologies respectively in their digital photos. The top-view of two membranes were continuous and no obvious cracks or pinholes could be found on the surface. Interestingly, the cross-section images of two membranes showed that dense and uniform COF layers with thickness of 2.0 ± 0.2 μm assembled on the supports, which were similar to the structural feature of thin (less than 1.0 μm) graphene oxide (GO) membranes[2,3]. We attributed this phenomenon to the high aspect ratio of TpPa-SO_3H and TpPa-CO_2H nanosheets. Owing to the moderate lamellar size, the nanosheets were easy to be rejected by the supports and had a well stack parallel to the supports, leading to the COF membranes with tight structures.

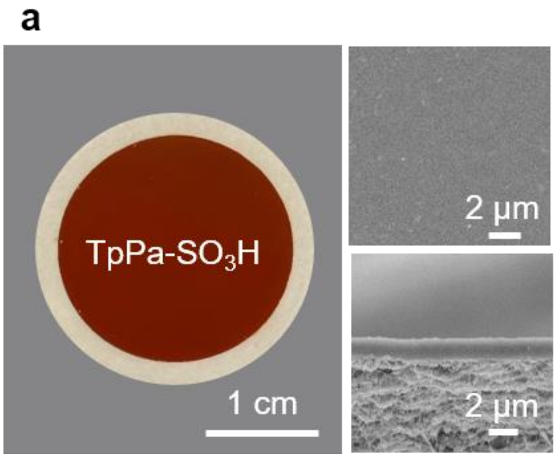

Supplementary Fig. 9 Morphologies of COF membranes. (a) Digital photo and SEM images of TpPa-SO_3H membrane. **(b)** Digital photo and SEM images of TpPa-CO_2H membrane.

3.2. ^{13}C ssNMR

The ssNMR experiments were performed on JEOL JNM ECZ600R with a frequency of 600 MHz, using a 3.2 mm Bruker MAS probe. For testing process, the COF nanosheets were prepared into self-standing membranes through a solution evaporation method: COF nanosheet colloidal was dropped into a teflon-made dish. The dish was transferred into an oven and kept static at 70 ^{o}C for 48 h. With the water evaporating from the dish, the self-standing COF membranes are successfully obtaiend. Subsequently, the TpPa-SO_3H, TpPa-PO_3H_2 and TpPa-CO_2H membranes were collected and grinded into powder samples for ssNMR experiments, as shown in Fig. 3a. Overall, ^{13}C ssNMR spectra for these COF samples matched well with the reference/predicted compounds(*1, 4*), and signified a similar skeleton (TpPa) environment. The differences of identified signal at ~185 ppm suggested the varied polarization strength of -SO_3H, -PO_3H_2 and -CO_2H bringing to the basic TpPa skeletons. Moreover, no obvious signal could be found in the range of 20-50 ppm for TpPa-PO_3H_2 membrane, which agreed well with the results of FTIR measurements, suggesting that no ethyl group remained in -PO_3H_2.

3.3. FTIR

FTIR measurements (MultiGas 2030 FT-IR spectrometer equipped with a ATR Diamant Golden Gate) were used to analyze the structural composition of COF membranes, as shown in Supplementary Fig. 10. With the absence of N-H (3335−3425 cm^{-1}) and -C=O (1650 cm^{-1}) stretching bands, it could be derived that the aldehyde and diamine monomers completely consumed or removed in the formed skeletons[4,5]. In addition, the absence of C=N bond (1620 cm^{-1}) and the presence of C=C bond (1569 cm^{-1}) showed that the nanosheets skeletons occurred enol-keto tautomer exchange process. The aromatic C=C and C-N bonds were identified according to the stretching peaks at 1427 cm^{-1} and 1248 cm^{-1} respectively, which further verified keto-tautomer structure in membranes. It was worthy to note that no obvious signal could be found at 2800-3000 cm^{-1} for TpPa-PO_3H_2 membrane, demonstrating that the ethyl groups existed in partially hydrated phosphoric acid diamines completely removed during TpPa-PO_3H_2 nanosheets formation.

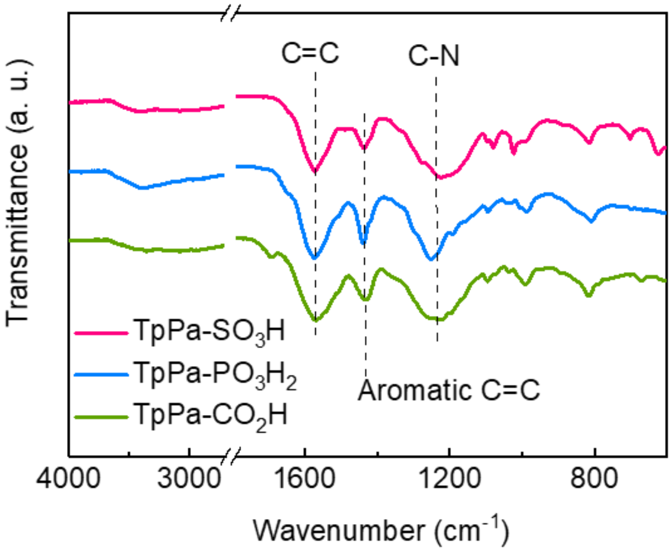

Supplementary Fig. 10 FTIR characterizations of COF membranes.

3.4. XPS

To gain insight into the compositions and interactions of COF membranes, XPS (ESCALAB Xi+) measurements were carried out using an Al-Kα microfocused monochromatized source with an energy resolution of 0.43 eV, spatial energy of 20 μm and a spot size of 400 μm. As shown in Supplementary Fig. 11, COF membranes showed functional groups (-C=O, -C-N and C-C) on the C 1*s* region (158-166 eV), which demonstrated the existence of keto-structures within membranes. This result was in good accordance with those of FTIR and ssNMR measurements. The elemental ratio estimated from XPS survey spectra showed that the content of -SO_3H and -PO_3H_2 was 1.86 at% and 0.80 at% for TpPa-SO_3H and TpPa-PO_3H_2 membranes respectively, suggesting the functionalization degree of TpPa-SO_3H and TpPa-PO_3H_2 membranes reached 1.86 at% and 0.80 at%, respectively (Supplementary Table 2). For TpPa-CO_2H membrane, due to that the functional group (-CO_2H) possessed the same elemental composition with TpPa skeleton, it was difficult to directly calculate the functionalization degree of TpPa-CO_2H membrane. Therefore, we treated the TpPa-CO_2H membrane in 1M NaOH aqueous solution tending to convert H^+ in -CO_2H into Na^+ to measure the content of -CO_2^-. In Supplementary Table 2, the content of Na element was estimated to be 0.96 at%. Assuming that H elements in -CO_2^- groups completely convert into Na^+, the functionalization degree of for TpPa-CO_2H membrane was thus around 0.96 at%.

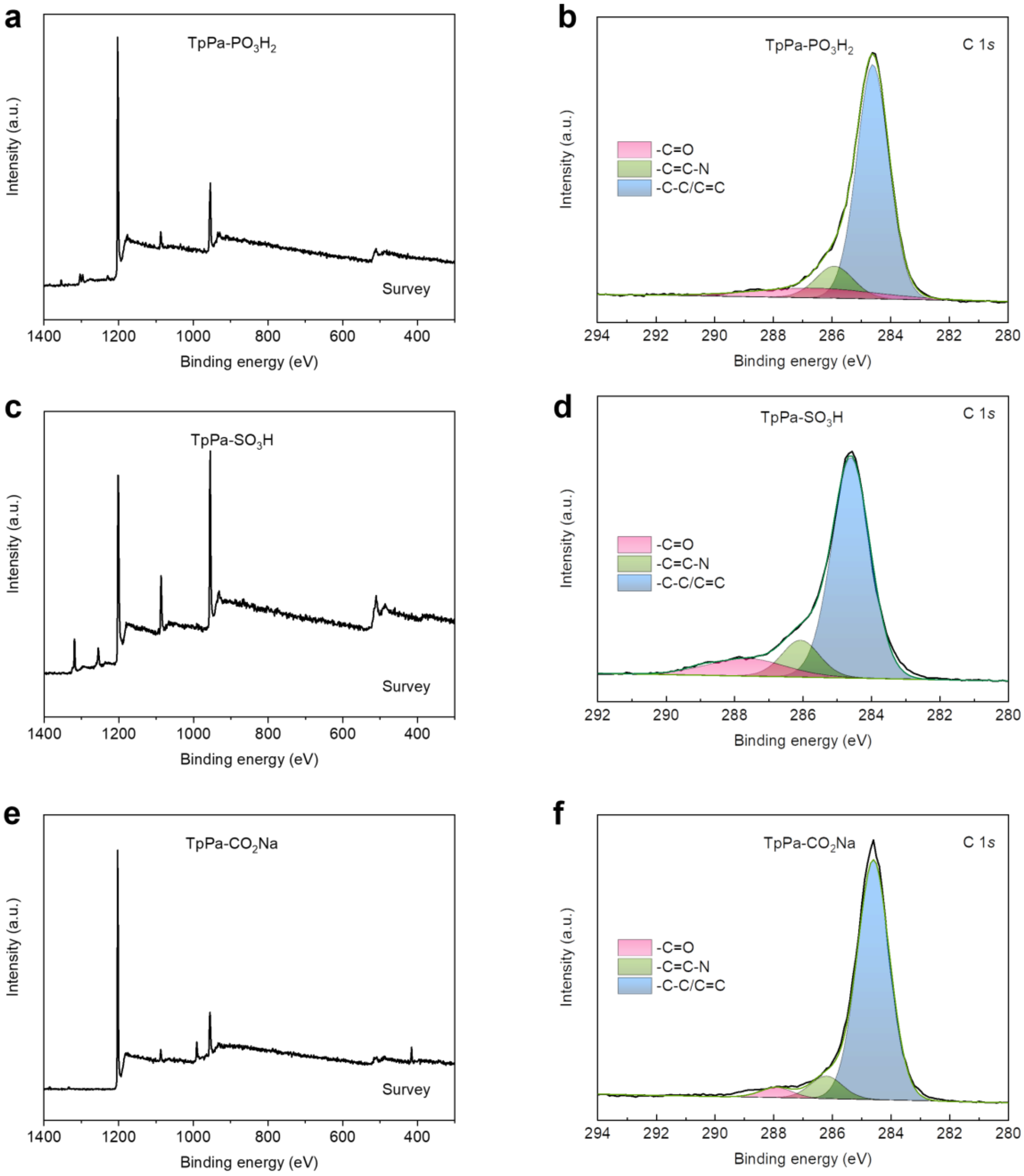

Supplementary Fig. 11 XPS results of COF membranes. Survey results and C 1*s* spectra for **(a-b)** TpPa-SO_3H membrane, **(c-d)** TpPa-PO_3H_2 membrane and **(e-f)** TpPa-CO_2H membrane.

Supplementary Table 2. XPS survey for COF membranes.

TpPa-SO_3H					
**Element**	**Peak position (eV)**	**FWHM (eV)**	**Area**	**At.%**	**Funct. degree (at%)**
**C**	284	2.59	2246926	76.90	
**N**	399	2.83	265194	5.29	
**O**	531	3.09	1306914	15.94	
**S**	167	2.69	130907	1.86	1.86

TpPa-PO_3H_2					
**Element**	**Peak position (eV)**	**FWHM (eV)**	**Area**	**At.%**	**Funct. degree (at%)**
**C**	284	2.71	2539029	83.52	
**N**	399	3.03	212272	4.07	
**O**	532	3.87	990356	11.61	
**P**	132	2.66	42530	0.80	0.80

TpPa-CO_2Na					
**Element**	**Peak position (eV)**	**FWHM (eV)**	**Area**	**At.%**	**Funct. degree (at%)**
**C**	284	2.32	2263541	88.31	

**N**	399	2.49	95257	2.17	
**O**	532	3.56	615627	8.56	
**Na**	1071	2.33	149025	0.96	0.96

3.5. Raman spectroscopy

Raman spectroscopy (LabRAM HR Evolution operating at 532 nm) measurements were performed to identify the composition of COF membranes. Generally, imine COFs had two main Raman modes, including the vibration of sp^3-hybridized amorphous C atoms of mode D and the in-plane stretching of sp^2-hybridized C atoms of mode G. From Supplementary Fig. 12, it can be seen that TpPa-SO_3H, TpPa-PO_3H_2 and TpPa-CO_2H membranes showed obvious signals at 1394 cm^{-1} and 1611 cm^{-1}. The observed signals corresponded to model D and model G, respectively, matching well with the results reported in literature[6]. Moreover, it was known that the ratio of I_D/I_G could be used to assess the defect degree of samples. In this work, the ratio of I_D/I_G for TpPa-SO_3H, TpPa-PO_3H_2 and TpPa-CO_2H membranes was estimated to be 0.78, 0.61 and 0.43, respectively, demonstrating that the disorder and defect degree gradually declined for TpPa-SO_3H, TpPa-PO_3H_2 and TpPa-CO_2H membranes.

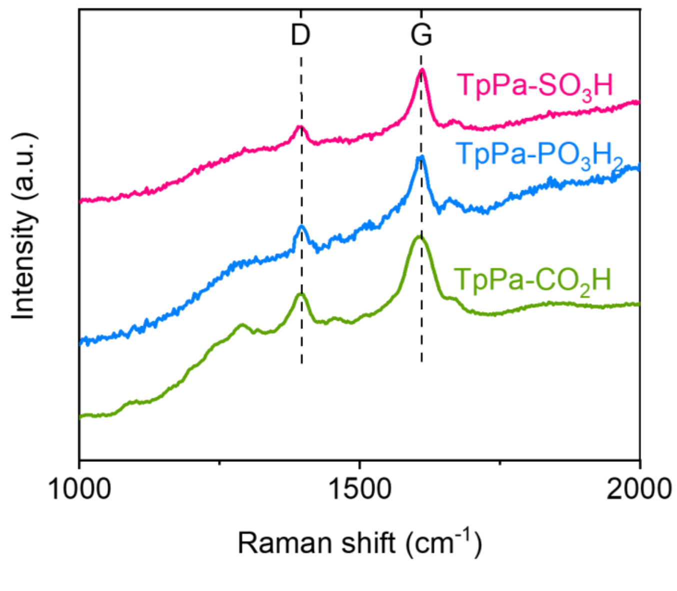

Supplementary Fig. 12 Raman characterizations of COF membranes.

3.6. XRD and 2D-GIWAXS

To identify the crystalline structures of COF membranes, X-ray diffraction (XRD) measurements were carried out using a using a RigakuD/max2500 v/Pc X-ray diffractometer (Cu Ka) in the 2*θ* range of 3-35°. For the sample preparation, COF nanosheets were filtrated on PAN supports with molecular weight cut-off of 100000. The resultant supported COF membranes were transferred into DMF to remove PAN supports and obtain self-standing COF membranes for XRD measurements. Fig. 3B showed three diffraction peaks appear at 2θ = 4.8°, 8.0°, and 26.8-27.2°, which were assigned to the (100), (110) and (001) planes[7] respectively for TpPa-SO_3H, TpPa-PO_3H_2 and TpPa-CO_2H membranes. The broaden peaks in XRD patterns were due to that small-sized crystals or crystalline domains existed in the COF membranes. Moreover, the (100) plane exhibited a moderate relative intensity compared with other planes, which suggested a higher degree of crystallinity for the COF membranes.

Two-dimensional synchrotron radiation grazing incidence wide-angle X-ray scattering (2D-GIWAXS) was performed at BL14B beamline, Beijing Synchrotron Radiation Facility with a wavelength of 1.54 Å to analyze the crystallinity and orientation of COFs membranes. 2D-GIWAXS data was collected *via* a MarCCD with a distance of 438 mm from the samples. From Fig. 3E, it can be seen that TpPa-SO_3H, TpPa-PO_3H_2 and TpPa-CO_2H membranes had a signal at q_{xy} = 0.35 $Å^{-1}$ corresponding to (100) plane. The concentrated signal of (100) plane along q_{xy} axis indicated that the 1D COF channel was perpendicular to the horizontal direction (or the surface of filtrated supports). In addition, the red signal of (100) plane also unveiled the high crystallinity of COF membranes, consistent with the results of XRD pattern.

3.7. Simulated stack model

The experimental data of TpPa-PO_3H_2 was collected using XRD measurements based on the synthesized powders. In a typical TpPa-PO_3H_2 powder synthesis procedure, 0.1 mmol of Tp, 0.15 mmol of Pa-PO_3H_2 and 3 mL of solvent mixture (dioxane: mesitylene: 3M acetic acid = 1: 1: 1) were added into a pyrex tube. The obtained mixture was sonicated for 5 min, and was flash frozen at 77 K (liquid N_2 bath) and degassed by three freeze-pump-thaw cycles. The mixture was then heated at 120 °C for 3 days. After reaction, the red precipitate was collected and purified with THF, acetone and methanol to remove any unreacted monomer or oligomer. The red product was then dried at 60 °C under vacuum for 24 h to give TpPa-PO_3H_2 powder with a ~46% isolated yield. Possible models were built and optimized employing the Self-Consistent Charge Density Functional Tight Binding (SCC-DFTB) method with London dispersion corrections. All possible relative positions of the -PO_3H_2 functional groups were optimized and the lowest energy configurations were used to simulate the PXRD patterns (Supplementary Table 3). From Supplementary Fig. 13, it can be observed that the simulated PXRD patterns of the slip-AA eclipsed stacking model matched well with the experimental PXRD patterns of TpPa-PO_3H_2 powder. In order to determine the unit-cell parameters, Pawley refinements of the observed PXRD patterns were also performed for TpPa-PO_3H_2, which revealed a highly ordered framework structure of TpPa-PO_3H_2 powder.

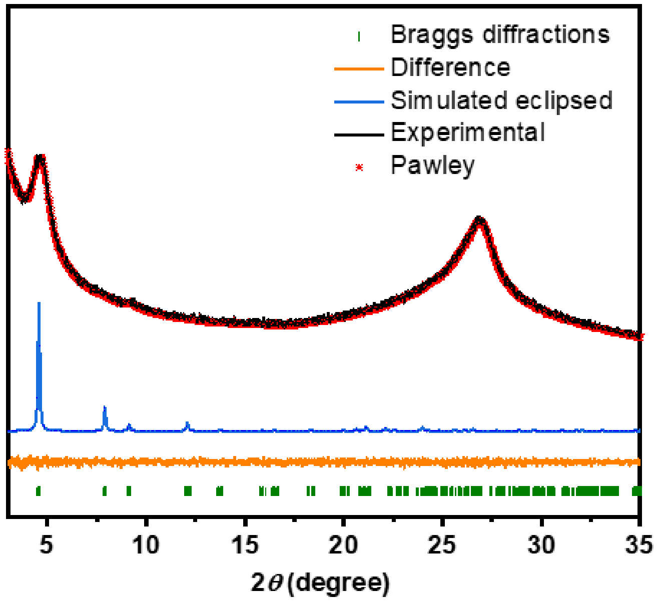

Supplementary Fig. 13 Experimental powder pattern and Pawley refined pattern of TpPa-PO_3H_2 based on PI symmetry.

Supplementary Table 3 Functional atomic coordinates for the unit cell of TpPa-PO_3H_2.

Parameter	TpPa-PO_3H_2<br>Triclinic; P 1<br>a=22.302 Å; b=22.3928 Å; c=6.8744 Å<br>α=90, β=90, γ=120		
Atom list	x	y	z
C1	0.19226	0.57039	0.59746
C2	0.63678	0.26431	0.64354
C3	0.70531	0.29546	0.5394
O4	0.53344	0.26892	0.75231
N5	0.85562	0.3998	0.47507
C6	0.92919	0.44016	0.42927
C7	0.97258	0.41799	0.55996
C8	0.04463	0.46069	0.54938
C9	0.44192	0.63205	0.51792
C10	0.73443	0.35887	0.37746
C11	0.69212	0.3863	0.29418
O12	0.74031	0.25856	0.57959
N13	0.6031	0.45328	0.39936
C14	0.55921	0.48319	0.42467
C15	0.58139	0.55084	0.31996

C16	0.53855	0.5796	0.35988
C17	0.37833	0.81627	0.59398
C18	0.62385	0.35686	0.41298
C19	0.59732	0.29658	0.5978
O20	0.71932	0.4413	0.08384
N21	0.544	0.14804	0.70418
C22	0.50885	0.07561	0.77545
C23	0.44245	0.03315	0.65038
C24	0.41139	0.96129	0.66918
C25	0.8086	0.39835	0.29149
C26	0.35796	0.74273	0.59517
C27	0.28702	0.69254	0.60229
O28	0.47916	0.76958	0.54436
N29	0.14767	0.57342	0.41323
C30	0.07444	0.52602	0.40974
C31	0.03147	0.54649	0.26597
C32	0.95935	0.50439	0.27715
P33	0.39842	0.07043	0.42684
C34	0.57984	0.38789	0.35402
C35	0.2663	0.62231	0.57709
C36	0.31618	0.6014	0.5518
O37	0.23577	0.71062	0.64635
N38	0.42803	0.56871	0.55112
C39	0.47473	0.54274	0.51373
C40	0.45356	0.47647	0.62205
C41	0.49528	0.44692	0.58041
P42	0.93659	0.33421	0.76059
C43	0.60599	0.19517	0.78461
C44	0.38738	0.65134	0.55225
C45	0.40811	0.72184	0.56823
O46	0.29622	0.53168	0.51352
N47	0.42043	0.85812	0.80327
C48	0.44572	0.93091	0.81544
C49	0.50859	0.97301	0.96534
C50	0.54047	0.04491	0.94532
P51	0.66248	0.60119	0.09928
O52	0.45111	1.13896	0.19133
O53	0.36167	1.09899	0.63388
O54	0.33292	1.01211	0.1892
O55	0.6628	1.66493	-0.12683
O56	0.72719	1.64195	0.30676
O57	0.68572	1.55636	-0.15033
O58	0.86699	1.31212	1.00191
O59	0.99611	1.32562	0.98399
O60	0.90967	1.2724	0.53354

H61	0.17669	0.53004	0.76911
H62	0.50906	0.29656	0.74577
H63	0.07648	0.442	0.65032
H64	0.49438	0.67253	0.47182
H65	0.7852	0.27718	0.45907
H66	0.55531	0.63016	0.26464
H67	0.35799	0.83407	0.40988
H68	0.68527	0.4551	0.00421
H69	0.36098	0.92987	0.56541
H70	0.82312	0.42906	0.08106
H71	0.49228	0.81981	0.53395
H72	0.05457	0.59664	0.15499
H73	0.92746	0.52348	0.17904
H74	0.52526	0.35361	0.30894
H75	0.25255	0.75947	0.71837
H76	0.40416	0.44811	0.74046
H77	0.47827	0.39628	0.67623
H78	0.63931	0.18271	0.91794
H79	0.24585	0.49903	0.46216
H80	0.53428	0.94944	1.08517
H81	0.59036	0.07541	1.05459
H82	0.97205	1.27582	1.03348
H83	0.83869	1.26152	0.97667
H84	0.42291	1.16055	0.1436
H85	0.30652	1.03567	0.13997
H86	0.71189	1.69283	-0.1908
H87	0.73572	1.58838	-0.18537

3.8. N_2 adsorption-desorption

In order to identify porosity and pore size distribution of COF membranes, N_2 adsorption-desorption experiments (up to 1 bar) were carried out using an automatic volumetric instrument. The preparation method of membrane samples was the same as that of ^{13}C NMR test. Supplementary Fig. 14 showed the results of N_2 adsorption isotherms collected at 77 K. It can be found that the COF membranes exhibited Type-I adsorption isotherms, confirming their microporous feature. The porosity of membranes evaluated by Brunauer-Emmett-Teller (BET) method gave surface areas of 107 m^2 g^{-1}, 379 m^2 g^{-1} and 160 m^2 g^{-1} for TpPa-SO_3H, TpPa-PO_3H_2 and TpPa-CO_2H membranes, respectively. Unlike the high porosity of reported counterpart COF powders, these membranes exhibited a lower porosity metric. This could be attributed to the higher aspect ratio of COF nanosheets. The angstrom-thick COF sheets made it difficult to adsorb abundant gas molecules during the experiments, leading to the lower surface areas compared with the powder counterparts. In addition, Fig. 3c demonstrated the pore size distribution of COF membranes concentrated on 13.3-13.4 Å, which was consistent with the XRD projection data and simulated pore metrics, thus further confirming the average pore parameters of COF membranes.

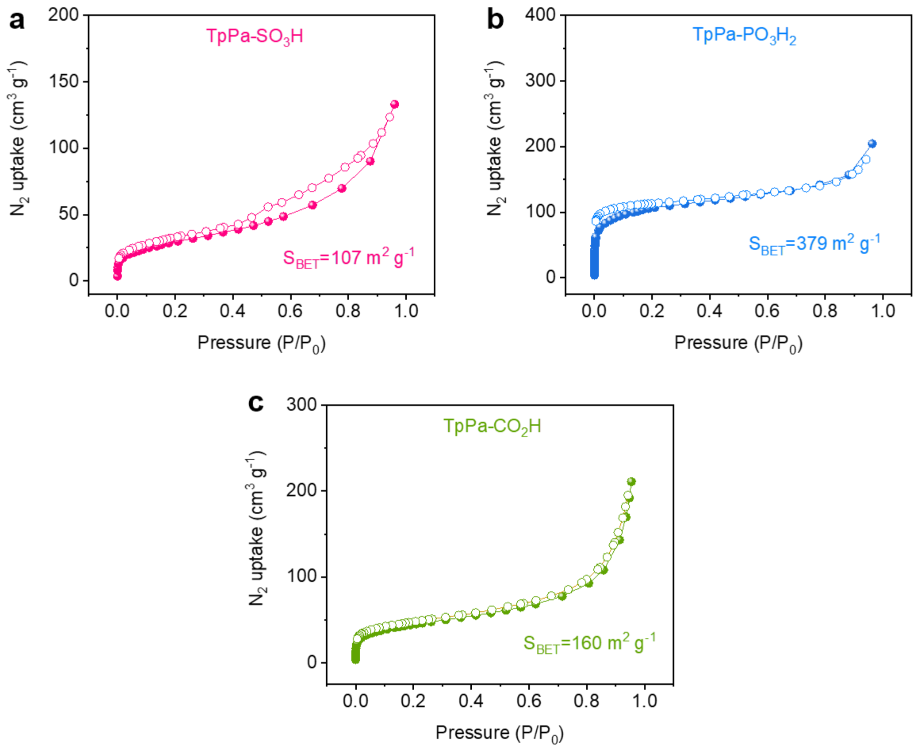

Supplementary Fig. 14 N_2 adsorption-desorption isotherm of COF membranes. (A) TpPa-SO_3H, **(B)** TpPa-PO_3H_2 and **(C)** TpPa-CO_2H.

3.9. Hydrophilicity

We used water contact angle to assess the hydrophilicity of COF membranes with varied functional groups. As shown in Fig. 3f, the water contact angle of TpPa-SO_3H, TpPa-PO_3H_2 and TpPa-CO_2H membranes was estimated to be 29.2°, 39.2° and 66.7°, respectively. The water contact angle of TpPa-SO_3H was close to the ones reported previously. The hydrophilicity difference of COF membranes could be mainly attributed to the varied acid groups, of which the hydrophilicity for -SO_3H, -PO_3H_2 and -CO_2H membranes followed a decline trend. Moreover, the water contact angle of TpPa-SO_3H and TpPa-PO_3H_2 was below 40°. This hydrophilic feature of TpPa-SO_3H and TpPa-PO_3H_2 enabled broad prospects for further applications of water treatment.

To assess the water adsorption ability of COF membranes, quartz crystal microbalance (QCM) measurements were carried out using Q-sense E1 supplied by Biolin Scientific Company (Sweden). For sample preparation, gold chips that were immersed into mixed solvents (DI water: ammonium hydroxide: hydrogen peroxide = 5 v: 1 v: 1 v) under 75 °C for 15 min. The gold chips were then dried with ultraviolet ozone condition to remove any residue. Subsequently, the gold chips were dropped with 50 μL COF nanosheet aqueous solutions (0.1 mg/mL) and dried under 40 °C for 12 h. For a typical measurement procedure, the chips with COF samples were placed into the testing module to confer a fundamental frequency under air condition. Then, the fluidic solution (DI water) with a rate of 0.1 mL min^{-1} flowed through the testing module to evaluate the adsorption behavior of COF materials toward water molecules. In our study, we selected the fundamental frequency (n=3) to investigate the change of frequency. The adsorption weight of COF materials toward the target species was calculated using Supplementary equation 2:

$$\Delta f = \frac{2f_0^2}{A\sqrt{\rho_q \mu_q}} \times \Delta m \quad (2)$$

Where Δf (Hz) was the change of frequency after the tests; Δm (mg) was the weight change on the surface of chip samples after the tests; f_0 (Hz) was the fundamental frequency; A (cm^2) was the effective area of chip samples; ρ_q was the density of quartz crystal (2.648 g cm^{-3} is this work); μ_q was the shear modulus of quartz crystal (2.947×10^{11} g cm^{-1} s^{-2} is this work). As shown in Fig. 3g, the capacity of water adsorption for TpPa-SO_3H, TpPa-PO_3H_2 and TpPa-CO_2H membranes was 8.96 mg/mg, 8.63 mg/mg and 7.26 mg/mg respectively and exhibited a decreasing trend. We attributed this phenomenon to the difference of hydrophilicity and relative surface area of three materials. TpPa-SO_3H and TpPa-PO_3H_2 possessed strong water constraint ability and high surface area, thus affording high water adsorption. In contrast, although TpPa-CO_2H had high surface area, the weak water constraint ability could not adsorb sufficient water molecules and form firm hydration shells in -CO_2H groups.

3.10. Charge property

Zeta potential measurements (ξ) of COF membranes were conducted using an equipment of Surpass analyzer operated under pH of 5.3-5.6. As shown in Supplementary Table 4, zeta potential for the COF membranes was negative and had an absolute value over 60 mV, indicating the presence of abundant negative charges in membranes. Moreover, zeta potential for TpPa-SO_3H, TpPa-PO_3H_2 and TpPa-CO_2H membranes was -131.2 ± 1.2 mV, -129.3 ± 3.1 mV and -64.0 ± 0.3 mV, respectively. The decline trend of zeta potential was mainly attributed to the decreasing degree of acid group dissociation within TpPa skeletons. Because the degree of acid groups dissociation under neutral condition followed the trend that -SO_3H > -PO_3H_2 > -CO_2H, the electrons in TpPa skeletons was inhibited after grafting weakly dissociated acid groups, thus lowering the zeta potential of COF membranes. This phenomenon agreed well with the trend of WCA and QCM measurements, where the water contact angle of membrane exhibited that TpPa-SO_3H < TpPa-PO_3H_2 < TpPa-CO_2H and water adsorption capacity followed the trend of TpPa-SO_3H > TpPa-PO_3H_2 > TpPa-CO_2H.

Supplementary Table 4. Zeta potential measurements of COF nanosheets. All the error bars in this table represent the standard deviation of the experiments.

Samples	Zeta potential (ξ, mV)
**TpPa-SO_3H**	-131.2 ± 1.2
**TpPa-PO_3H_2**	-129.3 ± 3.1
**TpPa-CO_2H**	-64.0 ± 0.3

3.11. Aqueous stability

Aqueous stability is an essential parameter for 2D nanosheets based membranes, which defines and puts constraint on the practical application of 2D nanosheets based membranes. Here, we immersed the free-standing COF membranes (with a thickness of ~2 μm) in water at 25 °C for one month to evaluate the aqueous ability of the membranes and gave a comparison among the commonly-seen 2D nanosheets based membranes. After immersing into aqueous conditions for one month, the COF membranes did not show macroscopic change or obvious macroscopic degradations. We assessed the swelling degree of COF membranes *via* the volume change before and after aqueous condition treatment, as shown in Supplementary Table 5. COF membranes only showed small swelling degree (<5%), much smaller than that of pristine GO membranes (60%-70%) and pristine MoS_2 membranes (5%-10%)[8]. This was mainly due to even-distributed functional groups of COF nanosheets, which conferred abundant interaction sites (H-bonding and van der Waals force) among adjacent COF nanosheets, thus fortifying the structural stability of COF membranes under aqueous condition. The swelling-resistant performance enabled COF membranes grand potentials for various separation applications under aqueous conditions.

Supplementary Table 5. Comparison of swelling degree for 2D nanosheet membranes. All the error bars in this table represent the standard deviation of the experiments.

Samples	Swelling degree
**GO**	60-70
**MoS_2**	5-10
**TpPa-SO_3H**	4.1 ± 2.3
**TpPa-PO_3H_2**	3.6 ± 1.8
**TpPa-CO_2H**	2.5 ± 1.0

3.12. Simulation of hydrated acid group structures

We further carried out simulation on the hydrated acid group structures. As zeta potential of -PO_3H_2 was slightly lower than that of -SO_3H (Supplementary Table 4), we set the condition as following: -SO_3^- had charge of -1e, -PO_3H^- had charge of -1e, and -CO_2^- had charge of -1e. In valence-charge, the charges of each atom were determined by the common valence of atoms. Normally, atoms had more charges compared to that from DFT calculations, leading more obvious hydration shells. In details, 1) O had charge of -1e, S had charge of +2e, so SO_3^- had charge of -1e; 2) O had charge of -1e, the –OH in PO_3H^- had charges of -0.5 for O and +0.5 for H; 3) O had charge of -1e, C had charge of +1e, so CO_2^- had charge of -1e. In valence charge model, the peak of solid blue line reflected that the interaction strength between acid groups and water molecules followed the trend of -SO_3H > -PO_3H_2 > -CO_2H. And the extreme point of dash blue line showed the radius of hydration shells for -SO_3H, -PO_3H_2 and -CO_2H was about 4.3 Å, 4.1 Å, 3.9 Å, respectively. Therefore, for individual channel/pore of the COF membranes, the acid group (-SO_3H and -PO_3H_2) could sufficiently and strongly bind water molecules, forming hydration shells to narrow the effective channel size

4. Ion transport performances of COF membranes

4.1. Ion permeation

Ion permeation measurements were performed using a glass-made diffusion cell (shown in Supplementary Fig. 15) purchased from Pomex company. For a typical ion permeation measurement, the compartments of diffusion cell were filled with 200 mL of DI water and 200 mL of chlorate salt aqueous solution, respectively. Supplementary Fig. 16 recorded the permeation rates of COF membranes under varied operation time. It can be observed that the COF membranes had stable permeation rates for different cations under 25 h, revealing the moderate operation stability of membranes.

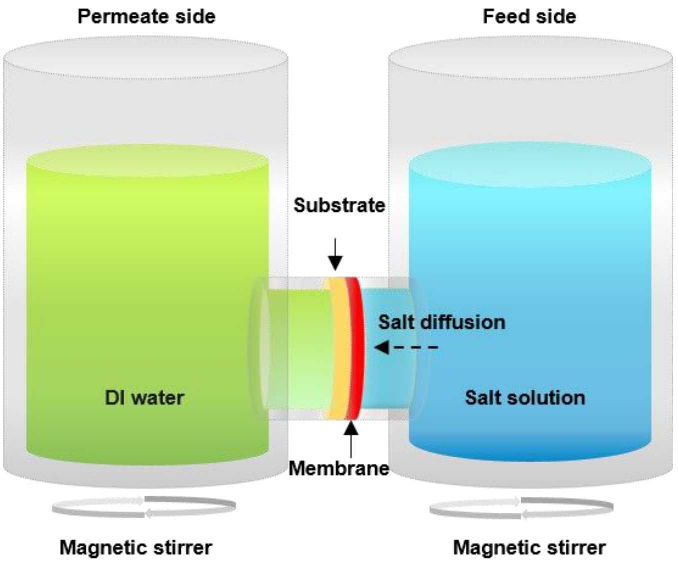

Supplementary Fig. 15 Diffusion cell used for ion permeation measurements.

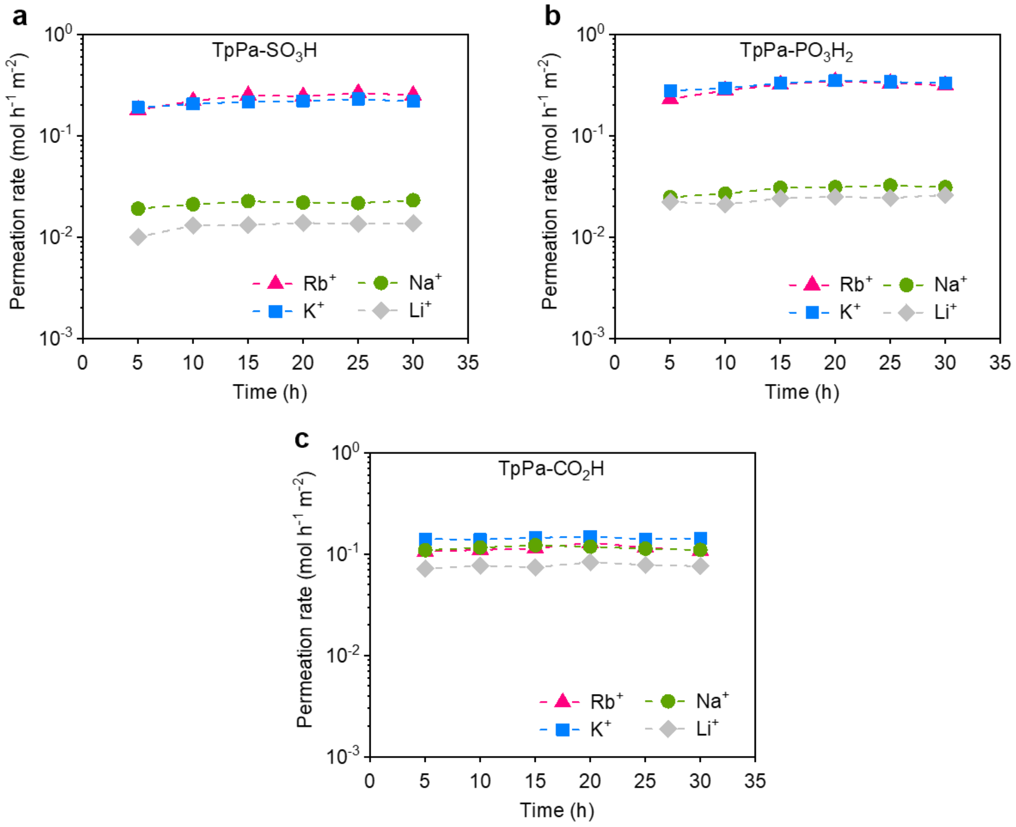

Supplementary Fig. 16 Ion permeation measurements of COF membranes under varied operation time. (a) TpPa-SO_3H membrane, **(b)** TpPa-PO_3H_2 membrane and **(c)** TpPa-CO_2H membrane.

4.2. Effect of hydration energy

We used TpPa-SO_3H, TpPa-PO_3H_2 and TpPa-CO_2H as example to investigate the impact of hydration energy of acid groups on ion transport, as shown in Fig. 5c and Supplementary Fig. 17. The channel size and acid group number of each membrane kept the same, but the group type was tuned by changing the hydration energy of each group. Based on the DFT calculation, the hydration energy for -SO_3H, -PO_3H_2 and -CO_2H is -0.44 eV, -0.36 eV and -0.15 eV, respectively.

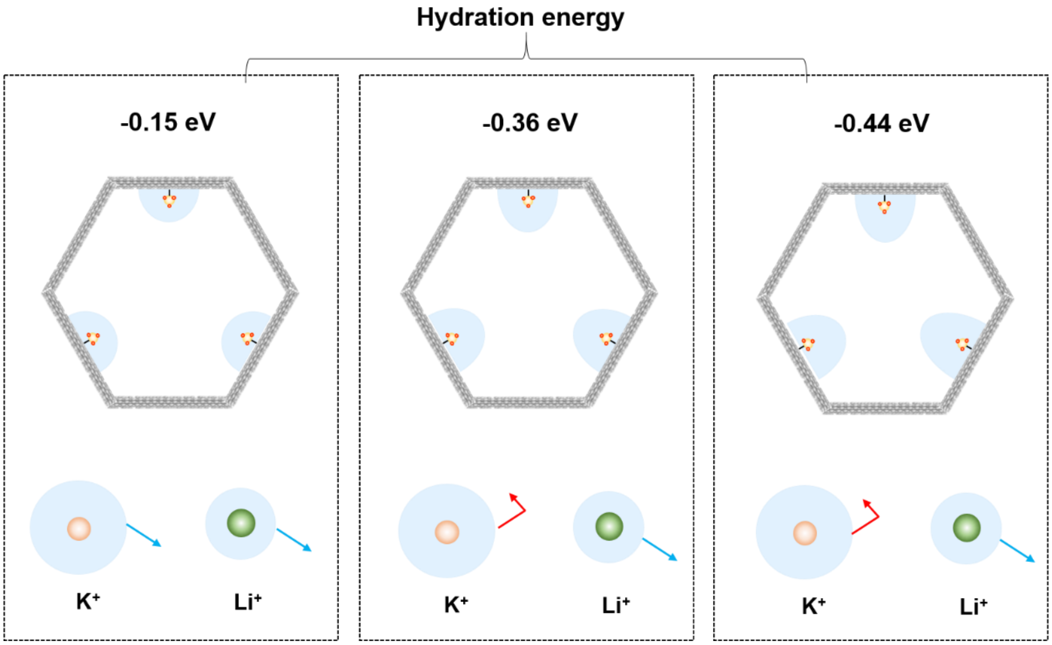

Supplementary Fig. 17 Schematic illustration of COF membranes with different hydration energy.

4.3. Effect of group density

In order to investigate the impact of acid group density on ion transport, we took sulfonic acid COFs as an example, keeping the same channel size the same while varying the number of -SO_3H group in each channel. 2, 5-diamine pyridine (Py) and 2, 5-diamine benzene-1, 4-disulfonic acid (Pa-$(SO_3H)_2$) were used as diamine monomers, respectively. The COFs nanosheets were prepared by three-phase interfacial polymerization (the intermediate layer of both COFs was 3 M aqueous acetic acid) and assembled respectively. The performances of TpPy and TpPa-$(SO_3H)_2$ membranes were compared with that of TpPa-SO_3H film, as shown in Fig. 5d and Supplementary Fig. 18.

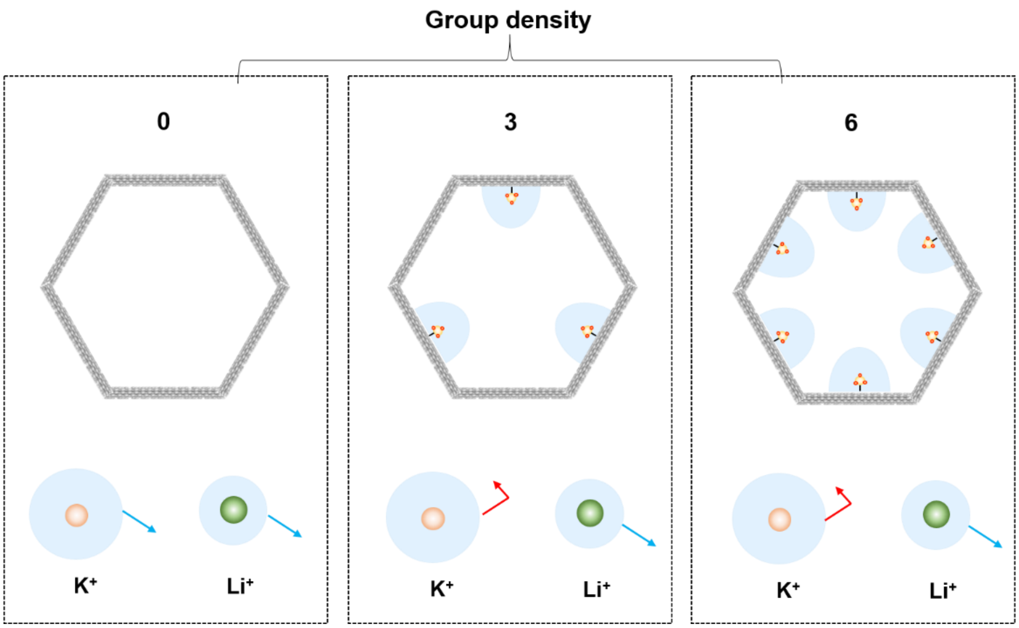

Supplementary Fig. 18 Schematic illustration of COF membranes with different group density.

4.4. Effect of group distance

We used sulfonic acid COFs as the template, and designed COFs membranes with different channel sizes to control the distance between groups. The COFs nanosheets and COFs films were prepared by three-phase interfacial polymerization using 2, 4-diamine benzenesulfonic acid (Ma-SO_3H) and 3, 3'-benzidine disulfonic acid (Bd-SO_3H) as diamine monomers, Tp as aldehyde monomers, and 3 M acetic acid aqueous solution as aqueous buffer layer. The performances of TpMa-SO_3H and TpBd-SO_3H membranes were compared with that of TpPa-SO_3H membrane, as shown in Fig. 5e and Supplementary Fig. 19.

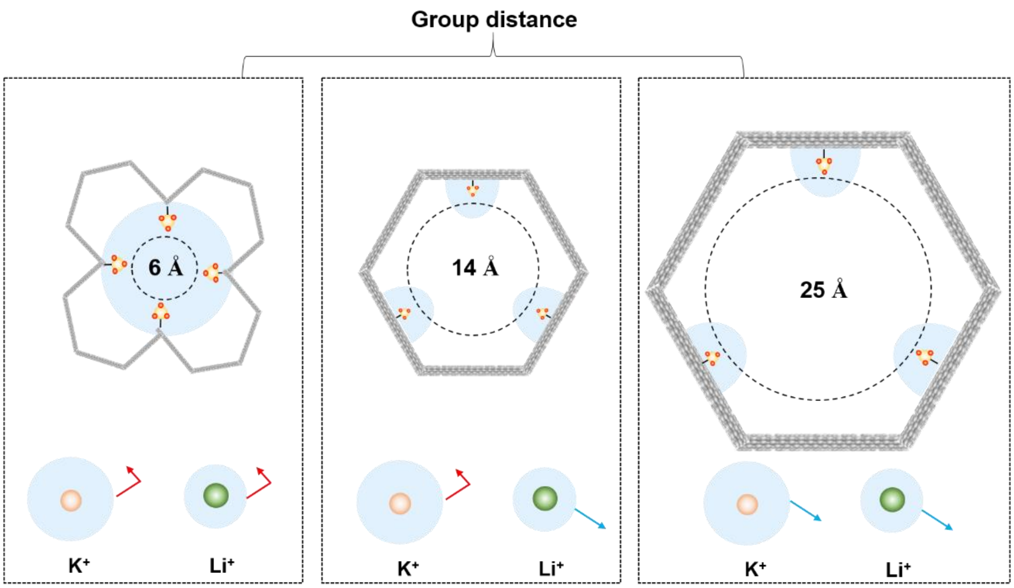

Supplementary Fig. 19 Schematic illustration of COF membranes with different group distance

4.5. Effect of ionic strength

In order to investigate the impact of ion diameter on ion permeation performance, we tuned the salt concentration in the feed side in the range of 0.01 M, 0.1 M, and 1.0 M. The diameter of K^+, Na^+ and Li^+ in water is 6.6, 7.1 and 7.6 Å, respectively[9-11]. Supplementary Fig. 20 showed the permeability of monovalent cations through the COF membranes under different ionic strength. The permeability of three cations increased with the increasing ionic strength. Interestingly, at lower ionic intensities of 0.01 M and 0.1 M, both TpPa-SO_3H and TpPa-PO_3H_2 membranes showed significant difference in permeability, of which the permeability of K^+ was about 5-16 times than those of the other two cations.With increasing ionic strength to 1.0 M, it can be found that the difference of permeability for TpPa-SO_3H and TpPa-PO_3H_2 membranes became smaller simultaneously. Meanwhile, the change of TpPa-SO_3H film was extremely significant, where the ratio (K^+/M^+) of ionic peemeability fall within 1.0 and 2.0. The change of permeability for the membranes with the varied ionic strength can be attributed to changes in the interaction between cations: at low ionic strength, the interference among cations was low, and the hydration diameter of ions was large. Therefore, the permeability process was significantly affected by the hydrated channel of COF membranes, reflecting high permeability difference among three cations. At high ionic strength, the interference among cations was strong and the corresponding ion hydration was directly reduced[8,12], which weakened the confinement effect of membrane channels and led to low permeability difference among three cations. In contrast, the TpPa-CO_2H membrane had a small difference in permeability under different ionic strength, indicating that the confinement effect of hydrated acid groups in channel was weak and it was difficult to achieve the selective trasport of monovalent cations.

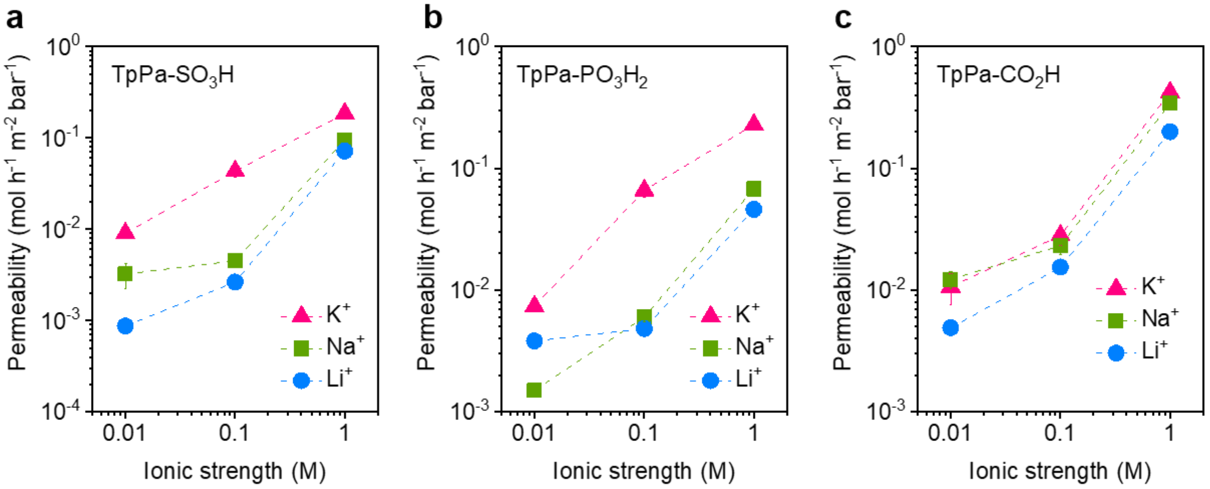

Supplementary Fig. 20 Permeability of cations across COF membranes under different ionic strength. **(a)** TpPa-SO_3H membrane, **(b)** TpPa-PO_3H_2 membrane and **(c)** TpPa-CO_2H membrane. All the error bars in this figure represent the standard deviation of the experiments.

4.6. Effect of electrostatic interaction

We investigated the influence of binding energy between cations and membranes on ion transport performances of COF membranes. Utilizing DFT calculations (performed with ORCA 13 at the B3LYP level and with the def2-TZVP basis set), we estimated the binding energy between cations (K^+, Na^+, Li^+) and two COF membranes, as listed in Supplementary Table 6. Supplementary Fig. 21 showed the permeability for COF membranes as a function of ionic binding energy. For electrostatic interaction dominated process, the larger ion-channel interaction energy was, the lower permeability of cations was through the membranes[9,10]. In this study, the permeability of K^+, Na^+ and Li^+ did not obey the above rule, i.e., the increase of electrostatic interaction did not lead to the decrease of ion permeability. Taking TpPa-CO_2H membrane as an example, its electrostatic interaction with K^+ and Na^+ was -3.65 eV and -7.03 eV respectively, while the corresponding ion permeability was about 0.028 mol h^{-1} m^{-2} bar^{-1} and 0.024 mol h^{-1} m^{-2} bar^{-1}, respectively. It further indicated that the electrostatic interaction of ion-membrane channel was not the decisive factor of ionic separation in the COF membranes.

Supplementary Table 6. Summary of the hydrated diameter, binding energy between K^+, Na^+ and Li^+ and the different functional groups.

Ions	Binding energy (eV)		
	**-SO3H**	**-PO3H2**	**-CO2H**
**K^+**	-3.44	-3.80	-3.65
**Na^+**	-6.80	-6.83	-7.03
**Li^+**	-4.67	-4.99	-5.09

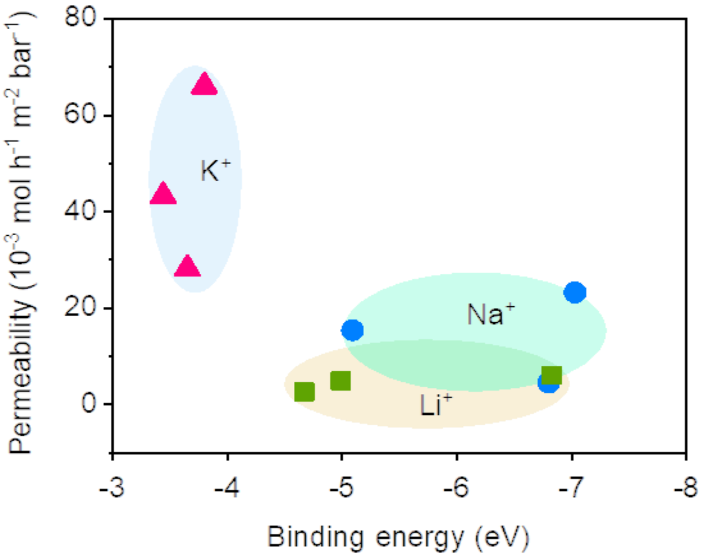

Supplementary Fig. 21 Permeability of K^+, Na^+ and Li^+ across COF membranes under varied binding energy between cations and membranes.

4.7. Effect of pH and temperature

Since the change of pH will directly affect the dissociation and hydration behavior of acid groups, and has a negligible effect on the cation in the solution, the influence of confined cascade separation on membrane performances was investigated by adjusting pH. We set the pH as 3.0, 4.2, 5.5 and 8.1, and obtained that the zeta potential of TpPa-PO_3H_2 membrane under the corresponding conditions was about -110 mV, -122 mV, -129 mV and -155 mV, respectively. As shown in Supplementary Fig. 24a, with the increase of pH from 3.0 to 8.1, the permeation rates of K^+ and Li^+ in binary system decreased from 0.18 mol h^{-1} m^{-2} and 0.050 mol h^{-1} m^{-2} to 0.13 mol h^{-1} m^{-2} and 0.028 mol h^{-1} m^{-2}, respectively. Conversely, the actual selectivity increased from 3.6 to 4.6. This result could be attributed to the amplification effect of confined stage. The stronger the hydration energy of acid groups the more stable the hydration structure was formed, and intensified spatial screening function toward binary mixtures. We also investigated the effect of operation temperature on separation performances. It could be observed that the actual selectivity had a slight decrease with increasing the temperature from 293 K to 323 K (Supplementary Fig. 24b), which was due to that the increasing temperature weakened hydration ability of acid groups and thus restricted the actual selectivity of membranes toward binary mixtures. The activation energy of K^+ and Li^+ was 10.94 kJ mol^{-1} and 13.01 kJ mol^{-1} for TpPa-SO_3H membrane, which was higher than K^+ and Li^+ for TpPa-SO_3H membrane (5.80 kJ mol^{-1} and 7.83 kJ mol^{-1}), as shown in Supplementary Fig. 24c-d. This result demonstrated that -SO_3H group was more sensitive than -PO_3H_2 group toward different temperature, which led to TpPa-SO_3H membrane had a more significant decrease of permeation rate with the increased operation temperature.

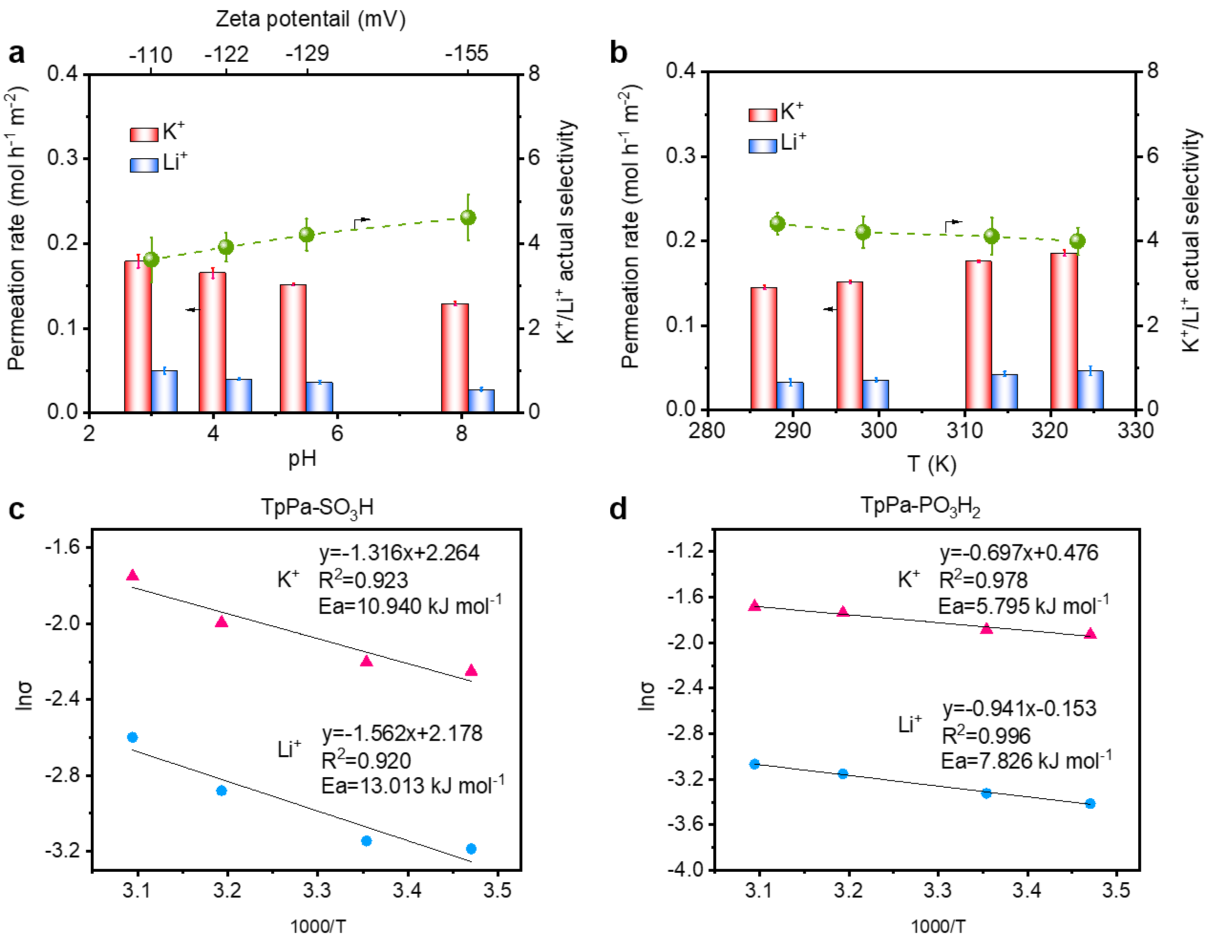

Supplementary Fig. 22 Separation performances for binary mixtures of monovalent cations within TpPa-PO_3H_2 membranes. (a) Separation performances of TpPa-PO_3H_2 membranes toward binary mixtures of monovalent cations under different pH. **(b)** Separation performances of TpPa-PO_3H_2 membrane toward binary mixtures of monovalent cations under different operation temperature. Activation energy of (**c**) TpPa-PO_3H_2 and (**d**) TpPa-SO_3H membranes measured from varied temperature, respectively. All the error bars in this figure represent the standard deviation of the experiments.

5. Supplementary References

1 Cao, L. *et al.* Weakly humidity-dependent proton-conducting COF membranes. *Advanced Materials* **32**, 2005565 (2020).

2 Liu, Y. *et al.* Graphene oxide membranes with an ultra-large interlayer distance through vertically grown covalent organic framework nanosheets. *Journal of Materials Chemistry A* **7**, 25458-25466 (2019).

3 Wang, S. *et al.* Graphene oxide membranes with heterogeneous nanodomains for efficient CO_2 separations. *Angewandte Chemie International Edition* **56**, 14246-14251 (2017).

4 Kandambeth, S. *et al.* Selective molecular sieving in self-standing porous covalent-organic-framework membranes. *Advanced Materials* **29**, 1603945 (2017).

5 Khayum, M. A. *et al.* Chemically delaminated free-standing ultrathin covalent organic nanosheets. *Angewandte Chemie International Edition* **55**, 15604-15608 (2016).

6 Cao, Y. *et al.* Lithiation of covalent organic framework nanosheets facilitating lithium-ion transport in lithium-sulfur batteries. *Energy Storage Materials* **29**, 207-215 (2020).

7 Peng, Y. *et al.* Mechanoassisted synthesis of sulfonated covalent organic frameworks with high intrinsic proton conductivity. *ACS Applied Materials & Interfaces* **8**, 18505-18512 (2016).

8 Ries, L. *et al.* Enhanced sieving from exfoliated MoS_2 membranes via covalent functionalization. *Nature Materials* **18**, 1112-1117 (2019).

9 Lu, J. *et al.* Efficient metal ion sieving in rectifying subnanochannels enabled by metal-organic frameworks. *Nature Materials* **19**, 767-774 (2020).

10 Zhang, H. *et al.* Ultrafast selective transport of alkali metal ions in metal organic frameworks with subnanometer pores. *Science Advances* **4**, eaaq0066.

11 You, X. *et al.* Metal-coordinated sub-10 nm membranes for water purification. *Nature Communications* **10**, 4160 (2019).

12 Chen, L. *et al.* Ion sieving in graphene oxide membranes via cationic control of interlayer spacing. *Nature* **550**, 380-383 (2017).
